# A social justice research agenda for multilingual assessment in diverse educational contexts

**DOI:** 10.1515/applirev-2025-0278

**Published:** 2026-04-07

**Authors:** Saskia Van Viegen, Sunny Man Chu Lau, Angel M.Y. Lin

**Affiliations:** Education University of Hong Kong, Hong Kong, China; Bishop’s University, Sherbrooke, Canada

**Keywords:** assessment, multilingual assessment, language assessment, bilingual education, EAL, ESL

## Abstract

Research and practice in bilingual education have contributed significant insights into teaching and learning from a multilingual stance. Despite these efforts, language and curriculum subject assessment continue to adhere to monolingual practices, not reflecting students’ dynamic language use and potentially reinforcing English dominance and linguistic hierarchies. Transforming education practice requires concomitant change in current monolingual approaches to assessment such that multilingual pedagogies can contribute meaningfully to equity and inclusion across K-12, post-secondary and adult education contexts. Empirical research and theory generation in what multilingualism in assessment looks like can address equity and social justice for bi/multilingual students in our diverse sociolinguistic contexts and the teaching and learning needs of Indigenous, racialized and other historically marginalized students. Engaging with this broad research and teaching issue, this narrative review synthesizes recent insights from empirical studies and conceptual scholarship in multilingual education and assessment. We identify the possible conceptual articulations between these two fields that lend themselves to further research, particularly inquiry into how educators can develop and extend multilingual approaches to assessment in bilingual education, to challenge traditional focus on achievement of standardized language norms and isolated skills.

## 1 Introduction

Research and practice in applied linguistics, specifically in the areas of bilingual education and Teaching English to Speakers of Other Languages (TESOL), have contributed significant insights into the rich linguistic repertoires of multilingual students, documenting collaborations with teachers to envision teaching and learning from a multilingual stance (e.g., Cenoz and Gorter 2022; De Costa et al. 2017; Garcia et al. 2017; Tian et al. 2020). Language and curriculum subject assessment in English-medium schools, however, tends to adhere to monolingual practices, not reflecting students’ dynamic language use. This disconnect risks sabotaging multilingual pedagogies, as transforming education practice requires concomitant change in current monolingual approaches to assessment. Indeed in our discussions with teachers and administrators, we have heard about, albeit their desire to implement multilingual pedagogy, the extent to which they have had to work around limited and limiting approaches to assessment, neglecting or repackaging students’ multilingual competence to fit monolingual norms and expectations. In the name of supporting student success, such efforts uphold English dominance and linguistic hierarchies under the guise of neutrality. This contradiction is evident in both popular discourse and curricular reforms that reduce language and literacy learning to achievement of standardized norms and isolated skills. Moreover, it splinters inclusive and equitable efforts in education, compelling an ethical and moral imperative for teachers and researchers alike to explore and generate alternative approaches to recognize, understand, document, and indeed validate student learning, multilingually.

Recognizing the colonial imprint of assessment, research across language testing, applied linguistics, and TESOL has begun to account for the fundamental legitimacy of bi/multilingual students’ complex, dynamic language practices. Toward these ends, recent scholarship has shed light on how to reconfigure epistemological traditions and methodologies of assessment in equitable ways to better align with the language and knowledge making evident in their local classrooms and communities. Currently, much empirical research on multilingual approaches to assessment is being conducted in disparate contexts, internationally. Bringing this cutting-edge work together, recent edited volumes and international journals have focused on multilingual assessment, helping to move it forward into new contexts of education (see for instance *Language Assessment Quarterly* special issue on the construct of multilingualism in language testing [Schissel et al. 2019a, 2019b; *Language and Education* special issue on education and multilingualism [Gorter 2017]; *Journal of Multilingual Theories and Practices* special issue on assessment innovations in multilingual contexts [Arias and Schissel 2021]). Recent monographs have contributed to articulating multilingual approaches to assessment (Dendrinos 2024; Gottlieb 2023), yet more contributions from across global locations and perspectives is needed, including reports of empirical studies conducted across situated contexts, and examination of multilingual assessment with an explicit focus on teachers, TESOL & teacher education.

Contributing to this effort, this narrative review offers an overview of recent literature relating to multilingual assessment, to guide future research in education that addresses assessment of language as a multilingual construct. We synthesize insights1Insights drawn from the symposium, Multilingual Approaches to Assessment and Education, hosted on May 27-28, 2021 [online] by Saskia Van Viegen, (York University, Canada) Sunny Lau (Bishop’s University, Canada) and Angel Lin (Simon Fraser University, Canada). The symposium featured invited scholars from diverse global locations to share insights from empirical work conducted in research-practice partnership in their unique educational contexts. Speakers included David Cameron (Toronto District School Board), Jasone Cenoz (Basque Country UPV/EHU, Spain), Jim Cummins (OISE/University of Toronto), Mélanie Ducharme (Centre de services scolaire de Laval in Québec, Canada), Fauve de Backer (Ghent University, Belgium), Durk Gorter (University of the Basque Country, Spain), Eunice Eunhee Jang (University of Toronto, Canada), Nicholas Limerick (Columbia University, USA), Constant Leung (King’s College London, United Kingdom), Mario E. López-Gopar (Universidad Autónoma Benito Juárez de Oaxaca, Mexico), Hieu Pham-Fraser (Richmond School District, Canada), Loretta Robinson (Naskapi Curriculum Department, Naskapi Nation of Kawawachikamach), Jamie L. Schissel, (University of North Carolina at Greensboro, USA), Elana Shohamy (Tel Aviv University, Israel), Stef Slembrouck (Ghent University, Belgium), Piet van Avermaet (Ghent University, Belgium), and Koen Van Gorp (Michigan State University, USA). from empirical studies and conceptual scholarship in both multilingual education and language testing and assessment, identifying conceptual linkages between the two fields and highlighting gaps that lend themselves to further research. While it may yet be too early to conduct a full systematic or scoping review on this topic, we offer a critical appraisal of the breadth of scholarship and emerging evidence and on multilingual approaches to assessment in education to illuminate and inspire a future research agenda on this topic.

### 1.1 Rationale & background

In the past few decades, critical perspectives in applied linguistics, TESOL and bilingual education research have generated theoretical and conceptual tools to challenge dominant monolingual approaches to curriculum and pedagogy. Researchers have documented how language and power manifest in educational settings, within long standing colonial histories of linguistic imperialism situated in multicultural and multilingual realities (Haque 2012; Kubota 2016). Significant research in language education highlights the positive contributions that students’ home languages can bring to education. Drawing on multilingual theories of language (for instance, translanguaging [García and Wei 2014; Thibault and Lin 2025], codeswitching [Grosjean 2008], plurilingualism [Moore and Gajo 2009], and translingual practice [Canagarajah 2011]), research studies have generated evidence of the ways in which multilingual perspectives contribute to counter systemic discrimination toward speakers of other languages, particularly those whose race, gender, sexuality, migration status and social class intersect and overlap to compound experiences of marginalization. Importantly, this research positions multilingualism as the norm and emphasizes the complex, dynamic language practices of multilinguals for scaffolding new learning, promoting metalinguistic awareness, developing biliteracy, and valorizing students’ cultural and linguistic identities (Lau and Van Viegen 2020; Leung 2013; Lin and Chen 2025). However, the monolingual bias in assessment remains almost untouched amidst the shift toward socially-engaged, heteroglossic approaches to language teaching and learning, necessitating a need for research in multilingual approaches to assessment and evaluation. Despite great motivation to engage in and experiment with multilingual pedagogical approaches, educators and researchers need clarity on the theoretical constructs of multilingual assessment as well as classroom-based research evidence and related professional development in theory and practice to continue to inform and support more equitable approaches to teaching and learning.

This opening is inspired by scholars in language testing and assessment who have problematized the monolingual construct of language, raising questions about what has underpinned development of assessment tools and resources in the educational context for use with bi/multilingual students in particular. Language assessments operationalize language proficiency constructs, often rooted in White, Anglo/Eurocentric and monolingual bias and language ideologies that may inadvertently exclude certain language varieties, registers, and usage (Leung 2023). Dynamic and fluid language use and flexible multilingualism tends to be overlooked. Similar critique has been articulated by language education researchers, who, working with teachers and students in educational settings, have documented the ways in which language standards and tests may reinforce linguistic hierarchies and neoliberal skills-based views of language teaching and learning (Kubota and Bale 2020; Lau et al. 2017; Shohamy 2022; Steele et al. 2022; Van Viegen and Jang 2021). Countering this conceptualization of language proficiency and communicative competence requires recognition of the over-representation of Whiteness in language standards that position other English varieties as deficient or interpret Indigenous, immigrant and racialized students’ creative, hybrid translanguaging practice as abject, wherein multilingual competence may be judged as interference in target language use (Flores and Rosa 2015; Garcia et al. 2021; Motha 2014; Smith 2022). Without such recognition, efforts toward assessing language competence in schools – whether in language or curriculum subject-area classrooms – might only maintain and reproduce marginalizing socio-economic stratification.

### 2 Multilingual (ML) assessment: a social justice research agenda

Multilingual (ML) assessment aligns with the most current understandings of language as a multilingual construct, reflecting the complex, dynamic language practices of bi/multilingual speakers and communities. We use ML assessment as an umbrella term to conceptualize the construct of multilingualism in assessment (Chalhoub-Deville 2019; Schissel et al. 2019a, 2019b), including assessment of plurilingual competence and plurilingual learners (Melo-Pfeifer and Ollivier 2025), and assessment through an interlingual and intercultural orientation (Scarino 2020), among others, that recognize the rich language practices of bi/multilingual students, facilitating the reclaiming and use of the full range of their linguistic repertoire to demonstrate their academic knowledge and literacy skills. This understanding is guiding more socially just and equitable approaches to assessment across various educational contexts, inspiring a comprehensive mapping of current efforts and new directions. From this perspective, we propose a social justice research agenda to problematize colonial legacies and current inequities in assessment practice, and guide inquiry and teacher education across multilingual education settings (K-12, post-secondary, adult education), organized into four lines of inquiry:

1. Understanding educational and social consequences of assessment
2. Rethinking fairness, equity, validity and reliability in assessments from multilingual perspectives
3. Engaging multilingualism in curriculum and assessment policy
4. Documenting emergent approaches to multilingual assessment

Obviously, these lines of inquiry offer but one way of looking, to generate new ways of thinking about assessment. Taking stock of and learning from interdisciplinary perspectives, including research in language testing, and different methodological approaches, from quantitative research on large-scale standardized assessments to case studies of classroom-based research with teachers, can yield alternative understandings of assessment. Below, we expand each line of inquiry, with the goal of weaving insights that can provoke a re-evaluation of current practice, all contributing to transforming language education and strengthening teachers’ development of assessment literacy.

### 2.1 Understanding educational and social consequences of assessment

Critical scholarship in language testing points to issues of fairness, namely the use of high-stakes monolingual English language tests for determining access to education, employment, and citizenship for multilingual people. This research emphasizes that language assessments thereby exert a strong influence on educational practice and student learning, demanding attention to the sociopolitical impact of tests and the consequences of their use. These understandings have been achieved through the contribution of consequential research in language testing (Chalhoub-Deville 2016; Messick 1989), that is, the study of the effects of tests and assessments on students, teachers, administrators and families. Scholarship in this domain facilitates consideration of the educational and social impact of assessment, not just effects on individuals.

For instance, in migration contexts, language tests for citizenship and immigration purposes have been found to stand in as *de facto* gatekeeping tools for “undesirable” immigrants who do not speak the dominant language or variety of the host country or whose language proficiencies are considered not up to the native-speaker standard for employment, education or residence (Shohamy and Pennycook 2019). Yet “not granting citizenship on the grounds of language”, as McNamara and Shohamy (2008) point out, “is a violation of basic human/personal rights to welfare, education and other social benefits” (p. 93). A special issue of the journal *Language Assessment Quarterly* (Shohamy and McNamara 2009) critiqued the use of language proficiency tests for migration purposes in a number of countries such as Estonia, Latvia, the UK, the USA, and Israel. Similarly, access to higher education is heavily regulated by monolingual testing regimes such as IELTS or TOEFL, which measure a very limited range of linguistic abilities of bi/multilingual learners which can discriminate against multilingual learners who operate multilingually in their everyday lives. As Kubota (2011) points out, language tests are “closely and complexly linked to a larger political, economic, and ideological terrain” (p. 258). These critical perspectives draw attention to the struggles of language-minoritized peoples and the need to address their experiences with language testing, particularly when the social consequences of these tests are high.

Testing is a social practice imbued with power dynamics that should be accounted for (McNamara 2012). For instance, rethinking core concepts in assessment validity, Shohamy et al. (2022) invite expanded understanding of tests, calling for a multilingual perspective on *what* gets measured, to include the full language repertoire, and *how* it is measured, to propose development of test texts and tasks in two languages, reflecting the translanguaging processes that bi/multilingual students use not only to learn and process content, but also in their lives. Recently, a newly added component emphasizes the *use* of tests, recognizing the unique consequences of testing for different groups of students, particularly for bi/multilingual students learning in an additional language. Informed by this lens, more research from a social justice-oriented approach is needed to problematize the apparatus of assessment, particularly the impact of monolingual assessments on multilingual students’ achievement and educational consequences. Shohamy’s scholarship (e.g. 2011) in consequential research has inspired a greater number of empirical studies which have documented how, when compared to monolingual students, multilingual students tend to receive lower test scores. For instance, illustrating the importance of measuring differences in student performance of students identified as English learners (ELs) on standardized assessments, several studies in the U.S., have examined factors that contribute to the closing or widening of achievement gaps (Goodrich et al. 2021; Kieffer and Thompson 2018).

Therefore, to better understand educational and social consequences of assessment, further research in TESOL needs to (1) examine how bi/multilingual students complete monolingual English tests and assessments, to identify whether and how they are constrained by the use of only one of their language resources in meaning-making and communicating academic knowledge; (2) document the sociopolitical impact and consequences of large-scale standardized monolingual language assessments on multilingual students; (3) examine impact of classroom-based curriculum assessment standards on multilingual students; and (4) support educators to understand how assessment governs access to and opportunity for academic, professional and social mobility for multilingual learners across all settings (early years, K-12, post-secondary, community-based and adult learning contexts).

### 2.2 Rethinking fairness, equity, validity and reliability in assessments from multilingual perspectives

The work of critical language testing scholars offers a socially grounded conceptualization of fairness, validity and reliability that can be useful to guide research into multilingual assessment. Working from interdisciplinary approaches, shaped by applied linguistics, language teaching, and educational and psychological measurement, these researchers have challenged traditional notions of validity and reliability in language testing, arguing that these criteria were historically conceptualized as neutral or objective through the lens of monolingualism (Leung 2022). They argue, for example, prevailing constructs in English language proficiency (ELP) testing, including high-stakes standardized tests for citizenship, training, employment and education purposes, often rely on “cognitive, generic, fixed conceptualization” which privileges a compartmentalized view of languages, ignoring the language user in context with their flexible, integrated repertoire (Chalhoub-Deville 2019: 474). At the same time, traditional language test design and norm-referenced interpretations of test scores provided little meaningful information to teachers and students, to critically assess their learning, cultivate meta-cognitive language awareness, and provide direction toward next steps in their multilingual development (Kunnan and Jang 2009).

Earlier studies on multilingual students’ performance on English standardized tests have drawn attention to the issues of not only fairness and equity but also validity and reliability. For example, in educational measurement, Canadian researchers Kim and Jang (2009) used quantitative methods to examine differential item functioning (DIF) in examination of a province-wide standardized English literacy test for students identified as L1 or English language learners, illuminating how reading in a second or additional language is mediated by vocabulary knowledge and may influence whether students can engage with the text using their full range of comprehension skills. In the United States, Winke et al.’s (2018) study showed that selected standardized English reading and writing tests administered to children identified as either native or non-native English speakers, though classified as psychometrically reliable and valid, did not accurately reflect ELs’ language abilities. Following up the test with an interview, the researchers discovered that it was not the learners’ lack of English skills but rather limitations in assessment literacy or age-related cognitive abilities that had hindered their responses. The authors suggested that pairing language tests with an interview can improve validity and reliability. Such studies have been important to generate empirical evidence that monolingual tests can miss the target when assessing multilingual learners, raising questions about construct validity or construct-irrelevant variance, that is, testing language skills that are unrelated to the intended assessment purpose, such as a math test designed to measure mathematical thinking ends up testing students’ ability to read and understand math problems.

Broadly, language testing researchers have called for more nuanced understanding of assessment data beyond test scores alone to enhance equity and validity, making space for mixed method research in language testing to provide richer insight into bi/multilingual learners’ experiences (Jang et al. 2014). In paying attention to these critically-oriented studies in language testing, we can derive insights for our work in multilingual school settings, to better understand multilingual students’ unique performance in assessment, create better supports and accommodations, and align assessment tasks and evaluations with students’ multilingual learning lives. This effort makes a case for research on more socially-situated use-specific models of assessment that are helpful to teachers (Arias and Schissel 2021; Tai and Wei 2024), as well as how to develop teacher assessment literacy, guiding what and how learning evidence from multilingual students can be collected, analyzed, and reported. Indeed, research and development of multilingual assessments can generate data not only on students’ performance on multilingual tests but also teacher and student perceptions and experiences of these approaches through questionnaires, interviews, focus groups and think aloud protocols, to illuminate the many contributions of multilingual tests beyond just the potential for higher test scores.

Attention to these issues has begun to inform policy development and change in internationally-recognized language assessment frameworks. For example, in the recent companion volume to the Common European Framework of Reference (CEFR), the framework used to guide development of language assessments in the European context, mediation was added as a new plurilingual competence for language and content learning, focusing on how learners mediate texts, concepts, and communication within and between languages (Piccardo and North 2019). The notion of mediation captures the process of negotiation and creation of meanings across languages, recognizing how language users can extract one or more messages from verbal, visual or aural texts and relay them to others in a different language or within a language variety (Dendrinos and Karavas 2024; Piccardo 2024). Assessment tasks can be designed with the intention to encourage learners’ agency, strategic processing, and reflection on their language and strategy use to mediate communication and understanding. These tasks that “embrace contingency, dynamic fluidity and multilingualism in interactional language use” (Leung 2022: 70) can better address reliability and construct validity issues that language testers have pointed out.

With the paradigmatic shift in understanding language as a holistic, multilingual construct, what constitutes fairness, validity and reliability in teachers’ assessment practice requires an urgent re-examination. Assessing bi/multilingual competence in the context of monolingual tests can embrace a broader conceptualization of language assessment practice that maintains learners at the center, reflecting authentic target language use of learners’ full repertoires (Seed 2020). Further empirical research on the assessment of mediation, examining learners at various levels of bi/multilingual competence and in diverse socio-linguistic and educational contexts, and elaborating teachers’ experiences of assessing mediation, is needed to build a body of research in this domain in TESOL.

### 2.3 Engaging multilingualism in curriculum and assessment policy

Studies on the use of large-scale assessments show that often they are not put to good use for feedback on educational improvement, and in fact, they have contributed to a broader neoliberal trend of accountability culture. For example, in Canada, Jang and Sinclair (2021) argue that Ontario’s EQAO (Education Quality and Accountability Office) provincial assessment program in literacy and math skills has become an accountability device rather than a means to provide diagnostic feedback for improved instruction. Any testing policy, as the authors argue, needs to stay organic in the sense that multiple partners should be engaged in ongoing conversations to ensure alignment with evolving educational needs. In the UK, large-scale standardized examinations such as the General Certificate for Secondary Education (GCSE) are norm-referenced summative assessments with limited to no reference to the classroom realities of “shifting linguistic diversity, multi-ethnic and multicultural complexity” (Leung et al. 2021: 300). Such research is critical to point out that standardized tests are typically designed for monolingual students, not recognizing that children and youth migrating to a new cultural and educational context must adapt to a different testing and assessment culture, with language proficiency mediating their learning and academic experience. However, the results from these large-scale assessments are often being used for high-stakes policy decisions about school and district performance. Rather than lumping students as a homogenous group, nuanced, disaggregated data for multilingual students are much needed to monitor student progress and identify factors that can promote learning achievements (Goodrich et al. 2021). The results of these studies are also critical to education reform, generating insights and implications that have a direct impact on the shape of education policy in their respective nation-state contexts.

Changes to assessment policy, to recognize and respond to the heterogeneity of school populations, requires policy-engaged assessment research that monitors and identifies needs of students across language backgrounds. Presently, very little research in assessment policy has considered multilingual students in their own right, thus developing assessment policy research along the lines of a multilingual perspective may better inform the interpretation and use of system-wide assessments for policy decisions related to curriculum, assessment, and access and availability of language resources and dual language or bilingual programs. Data from nation or district-wide assessments should also be mapped onto concomitant policy changes in education. For instance, Menken (2008) documented the impact of No Child Left Behind mandates for high-stakes testing of multilingual language learners. These English assessments were unfair because students were being tested in a language they were still in the process of mastering, which penalized these students and their schools. Identifying possible mismatch between curriculum and assessment policy could inform program reforms and importantly TESOL teacher preparation and certification requirements for teaching in multilingual contexts, dual language program availability, among others.

Examining a new multilingual education policy and assessment procedures that used bilingual (Hebrew-Russian and Hebrew-Arabic) versions of a national science test for secondary students in Israel, Shohamy et al. (2022) found that some students who received bilingual versions of a science reading test received significantly higher reading comprehension scores than students in control groups that received monolingual tests. Think-aloud protocols illuminated students’ strategy use, finding that students used both languages efficiently, and student perceptions of the bilingual tests were positive and contributed to student well-being during testing. Importantly, the researchers concluded that bi/multilingual students engage their full linguistic repertoire in processing content, which has been demonstrated in the long history of system-wide approaches to assessment in South Africa (Heugh et al. 2017). Longitudinal data from state-wide trilingual math tests showed how students utilized their multilingual repertoire to perform in these high-stake exams, countering the view that large-scale assessment cannot offer meaningful diagnostic information about bi/multilingual students’ learning and achievement. Such research demonstrates the promising use of multilingual resources to provide a fairer method of assessing knowledge for language learners who cannot fully demonstrate academic learning in the language of instruction. More studies like this, across diverse educational contexts, can show the value and impact of bilingual tests in standardized assessments.

While research on assessment policy can provide insight into the consequences of assessment for multilingual students, it can also be enriched by examining curriculum policy that informs local language assessment practice. Across diverse educational jurisdictions, locally-developed language assessment frameworks have been articulated in curriculum policy as resources for teachers to document, monitor, evaluate and report on bi/multilingual students’ language learning. These frameworks are generally mandated or recommended by state or provincial education departments, including, for instance, EAL Assessment Framework for Schools (UK) (Leung et al. 2021), WIDA (USA) (2012) or Steps to English Proficiency (STEP) (Canada) (Ontario Ministry of Education 2015). These frameworks often comprise descriptor scales articulating a progression of language proficiency across distinct skills (reading, writing, oral communication), which both rely on and facilitate teacher judgment of students’ observable linguistic performances in the classroom. Research has shown that these local language proficiency frameworks support diagnostic, formative and summative purposes of assessment, promote teacher knowledge of and professional learning concerning language teaching and learning, and contribute pedagogic washback, thereby comprising a powerful influence in the classroom (Spiliotopoulos et al. 2024; Stille et al. 2016. Nonetheless, these frameworks too are developed from a monolingual orientation, based on teacher perspectives of language norms that tend to disregard students’ multilingual competence and languaging practice. Further research in TESOL on the relationship between assessment policies and practice will provide additional insights to what examining system-wide assessment data alone does not provide (Kieffer and Thompson 2018).

### 2.4 Documenting emergent approaches to multilingual assessment

There has been growing research studies, across the fields of language testing, applied linguistics, and language education, that has explored and identified various approaches to multilingual assessment (e.g., De Backer et al. 2017; De Backer 2019; Gorter 2017; Heugh et al. 2017; Leung 2013; Liddicoat and Scarino 2024; Menken and Shohamy 2015; Rea-Dickins et al. 2013; Schissel et al. 2018). It is important to document, take stock, and disseminate these emergent approaches widely to support and inspire local implementation or adaptation and transform teacher education. Broadly, current approaches can be understood along a continuum (see for instance, Shohamy 2011) that invites use of two or more languages in assessing learners’ knowledge of language and content on the one hand, and holistic, dynamic integration of learners’ languages/varieties with open borders on the other (Cenoz and Gorter 2022). This continuum echoes the current theoretical orientation of multilingual or cross-linguistic perspectives on translanguaging (i.e. Cummins 2021; MacSwan 2022) and unitary or holistic translanguaging (Wei and Garcia 2022). Below, we articulate a short compendium of relevant approaches, in both language and other curriculum content areas, and call for further research in light of pragmatic considerations of what is possible in multilingual classrooms.

Addressing the assessment needs of multilingual learners often stays within the scope of *test accommodations*, such as lengthening time allowance or allowing use of word-for-word dictionaries or glossaries. Other bilingual options include use of interpretation or transcription, often used for initial diagnostic purposes, reading tests, or exams in content areas such as science or mathematics. As Shohamy et al. (2022) argue, accommodation practices have been envisioned as temporary solutions to scaffold learning in the target language for language minority students. Test accommodations, however, can position learners’ multilingualism as potentially contributing to measurement errors (Abedi 2009; Rea-Dickins et al. 2013; Schissel 2014; Stansfield 2011) rather than as integral to assessment design (Solano-Flores et al. 2011). It is imperative to go beyond mere test accommodations to develop multilingual assessments oriented toward more integrated, holistic approaches that connect assessment with learning and counter the gatekeeping function of most language tests.

Often, teachers may be unfamiliar with connecting assessments or test items with clear curriculum learning objectives. Such connections are crucial to provide evidence of what bi/multilingual students understand and what needs to be improved and help illuminate their learning needs, for a more *learning-oriented assessment* approach (Saville 2019). Teachers can allow students to use their L1 or to translanguage during assessment tasks, eliciting their performance and allowing them to display their curriculum knowledge without adhering to strict language boundaries, especially for students in the beginning stages of learning in a new language. This kind of multilingual approach can support *diagnostic* and *formative purposes of assessment* to differentiate students’ language and content knowledge, illuminating what students know and can do, and whether they may be struggling with comprehension or their decoding or other language skills. Learning-oriented assessment provides *feedback* and *feed-forward* (Hamp-Lyons 2017) for both teachers and students to evaluate, plan and act on their teaching and learning in an ongoing manner.

In *classroom-based assessment*, education researchers have written about the use of *language portraits* and other forms of identity texts as a diagnostic resource for understanding students’ linguistic repertoires, language trajectories, and language learning needs (Kyrligkitsi and Mouti 2023; Prasad 2020; Soares et al. 2020; Stille and Cummins 2013). In dual language bilingual education contexts, researchers have proposed careful *translanguaging documentation* of multilingual students’ lived experiences and identities in order to inform ongoing instruction and assessment designs (Sanchez et al. 2018). This translanguaging documentation can provide a fuller diagnostic picture of how students perform holistically through their trans/languaging practice, particularly how they use languages for classroom communication and academic learning regardless of the appropriateness of the language features; what they know; and whether they can express concepts using features associated with one language or the other. Another example of translanguaging documentation takes the form of *learning stories* (Escamilla et al. 2024), an Aotearoa New Zealand philosophical approach (Carr and Lee 2019) to document children’s ongoing multilingual, multimodal practices inside and outside of school. Teachers use their own (or those provided by children’s families) observation notes, photos and/or other artifacts to create multimodal stories that capture children’s multilingual identities, practices and development. Researchers in Indigenous language revitalization contexts have documented similar action research projects with families and communities to make visible Indigenous lifeworlds and connect valued language and literacy practices in home and school for learning (see for instance, Aitken and Robinson 2020; Patrick et al. 2013). These documentations of students’ multilingual identities and resources function as learning-oriented diagnostics in the classroom, and continued documentation of and research into these approaches is integral to developing emic perspectives on fair, equitable, and practical approaches to assessment.

Focusing on CLIL classroom contexts in Hong Kong, Jiang et al. (2024) engaged educators in integrating multimodal and multilingual perspectives into assessment activities at the secondary and post-secondary level for translanguaging assessment. Specifically, they explored *digital multimodal composing* (DMC), that is, tasks requiring students to combine linguistic and other representational modes to display their knowledge using digital technologies. Students’ L1 was regarded as a multimodal meaning-making resource alongside English, images and other semiotic modes, and educators developed an assessment rubric to document students’ translanguaging competences across their DMC activities. This documentation was useful for evaluation purposes, providing evidence of students’ developing content knowledge, and eliciting their creative thinking. However, DMC was challenging for some students, which the researchers noted could be a source of construct-irrelevance and interfere with the validity of assessment. Researchers in other language programs (e.g., Chinese immersion in the US) have conducted similar explorations, integrating translanguaging into formative assessments to gain insight into students’ emerging bilingualism and support instructional decisions for gradual release of responsibility to the students (Wong and Gomez 2024).

Evidence-based insights on learning-oriented assessment of multilingual competence can be generated by and incorporated into not only classroom-based assessment but also *large-scale assessments*, generating information about students’ learning profiles. At the same time, new digital technologies can support learning-oriented assessment, and with help from AI tools, can provide automated evaluation and scoring, generate learning analytics, and deliver personalized and real-time feedback to students, prompting them to plan, monitor, and reflect (Chapelle and Voss 2017; Jin and Fan 2023). More applied linguistics and education research can engage this scholarship to support development and implementation of multilingual assessment practices.

## 3 Conclusions

Despite the growing knowledge base in this domain, teachers often ask, “what about assessment”? This question signals the need for greater evidence of whether and how multilingual approaches to assessment materialize in educational practice, as well as a need to synthesize and mobilize this knowledge for educational use. In this narrative review, we have focused on four themes relevant to language education, both to illuminate critical insights and inspire future research. First, following from Shohamy and Pennycook (2019), we suggest the need to address both test use and social consequences, mapped upon a wider agenda for social change, to not only critique, but to intervene in and address the inequities and discrimination facing multilingual students in testing and assessment practice, to develop tests that better reflect multilingual language use. These interconnections can reveal the power of assessment to define what counts as language or knowledge in schools, shaping not only curriculum and pedagogy, but also the relationships and interactions among students and teachers. Second, rethinking what constitutes fairness, equity, validity and reliability in assessment, can shed light on the deep-seated monolingual and sociocultural biases inherent in traditional forms of assessment (Leung 2022). The realities of multilingual classrooms demand a more socially grounded conceptualization of these notions, to underpin redesign of tests and assessments from a multilingual stance. Third, further research is needed to better inform assessment policy and curriculum designs from a multilingual perspective. Such work can illuminate how bi/multilingual students utilize their entire linguistic repertoire in unique ways in reading and writing processes to display their knowledge and bi/multilingual competence, in both language and non-language curriculum content areas. Helping to shape education to respond to the heterogeneity of student population, both classroom-based and large-scale assessments need to address the nuanced and diverse needs of multilingual students. Finally, while a diversity of approaches to multilingual assessment have been documented, more research in this domain is needed to support the development of classroom-based assessments that recognize multilingualism and linguistic varieties. Situated research underscoring assessment *for* learning (not just *of* learning) is needed to provide ongoing evidence of learning for teachers and learners as feedback and feed-forward (Hamp-Lyons 2017) to guide their ongoing goal setting, planning and acting across the full spectrum of educational settings and contexts. This research demonstrates that the reconceptualization of language as heteroglossic requires focused attention to assessment to ensure alignment across teaching, learning and evaluation to achieve intended equity goals.
